# Supplementary material for: Learning curve and surgical outcome of robotic assisted colorectal surgery with ERAS program
Source: Sci Rep. 2022 Nov 29;12:20566. doi: 10.1038/s41598-022-24665-w (PMC9709162; doi:10.1038/s41598-022-24665-w)
Supplement: Supplementary file 2 — Supplementary Information 2. [file 41598_2022_24665_MOESM2_ESM.zip › RAL Raw data-Ñ[▒K/072/OP_ñw╝╨░O▒Kñσ.pdf]

正式摘要(電子簽章：000000214282980)

請選擇...reporter

臺中榮民總醫院

Operation Note

[redacted]

- 手術日期：2021/04/08
- 手術醫師：VS 蔣鋒帆，CR 林陳彥，CR 楊曙榮，R 陳威遠，R 陳琬清
- Anesthesia : GE
- Position : Lithotomy
- Aseptic agents : Betadine Aqua , Zephnan

Diagnosis

- Pre-operative diagnosis :Suspicious mass lesion over para-rectum area
- Post-operative diagnosis : s/p robotic low anterior resection

Surgical Indication

- For removing the suspicious mass lesion

Operation

- 1. Robotic (XI) assisted low anterior resection
  2. Intraoperative colonoscopy : (-).
  3. High ligation (-) : IMA was not approached.
  4. Take down from the splenic flexure : (-)
  5. Robotic EndoGIA (45-4.3) X2 + EEA (33mm) were applied to complete the end-to-end anastomosis
  6. Proximal cut end: 5 cm, distal cut end: 2 cm
  7. A 12 Fr. penrose drainage tube was inserted from RLQ of abdomen into the pelvic
  8. ADHESION BARRIER FILM was placed below the central extended wound

Finding

- 1. A 5 x 4 cm tumor at para-rectum area. It may be originated from the rectum ?
  2. Severely adhesion was noted below the umbilicus, which was compatible with the hx of s/p laparoscopic operation for ruptured appendicitis
  3. No other gross tumor nor polyps was found
  4. There was no obvious visible liver metastasis, no visible peritoneal seeding
  5. No obvious ascites was noted
  6. Bilateral doughnuts : intact ; Air leak test: Done and no air leak was found

Procedure

- 1. General endotracheal anesthesia was induced and then the patient was placed in modified Trendelenburg lithotomy position.
  2. Preoperative antibiotics were given. A Foley catheter and nasogastric tube were placed.
  3. The abdomen was prepped and draped in standard sterile fashion.
  4. A small vertical incision was made above the umbilicus and a 8 mm trocar was inserted into the peritoneal cavity by open method. Peumoperitoneum was established with CO2 inflation. Operative findings were as mentioned above.
  5. Laparoscopy was inserted and then other two 8mm trocars were inserted over LUQ, RLQ of abdomen. Adhesion lysis was performed first to create space for the remaing trochars. Then, another 12mm trocars was inserted over RLQ area, and the other assist-port 5mm troca was inserted on RUQ.
  6. The IMA was recognized but remained untouched. Distal S-colon was partially freed after lysis the adhesion band.
  7. The colon was mobilized from retroperitoneum overlying the sacral promontory and into the pelvis.

- 8. The incision was then extended laterally and medially across the peritoneal reflection.
  9. The middle to distal rectum was divided with robotic endoGIA (45-4.3) X2.
  10. The umbilicum area wound of abdomen was further extended. We approached into the peritoneal cavity by open method. Distal S-colon and rectum was pulled out.
  11. The distal s-colon was then taken out for carefully inspection to make sure it has an excellent blood supply. The mesenteric fat was gently cleared from the terminal 2-3 mm of the bowel ends.
  12. The field was isolated with moist pad and the bowel opened and sized. It was determined that a 33 mm circular stapler could be accommodated. The tumor-containing tissue was removed. Then the anvil was inserted, followed by the purse string suture. The colon was then put back to the abdominal cavity.
  13. The anal canal was irrigated with distil water and then the circular stapler was introduced through the anus.
  14. The proximal bowel with anvil in place was engaged with the circular stapler and the stapler was then fired. Two complete donuts were obtained and the distal one was submitted to pathology for margin confirmation.
  15. The anastomosis was tested for leaks with irrigation solution and air introduced through the rectum and was found to be patent and intact.
  16. The bleeder was checked thorughly again.
  17. A 12 Fr. penrose drainage tube was inserted from RLQ of abdomen into the pelvic
  18. The trochar fascia wounds were all then closed with interrupted sutures via 1-O Vicryl.
  19. ADHESION BARRIER FILM was placed below the central abdominal wound.
  20. The extended fascia wound was closed with interrupted sutures via Num.1 Vicryl.
  21. Massive N/S was applied to irrigate the wound.
  22. The wound was further closed with 3-O Vicryl, and the skin was applied with SurgiSeal, then covered with Medipore.
  23. The patient tolerated the procedure well and was brought to the POR in stable condition.
- Estimated blood loss: minimum
- 器械數量: 正確
- 紗布數量: 正確

Image

- PAINT
  - abdomen

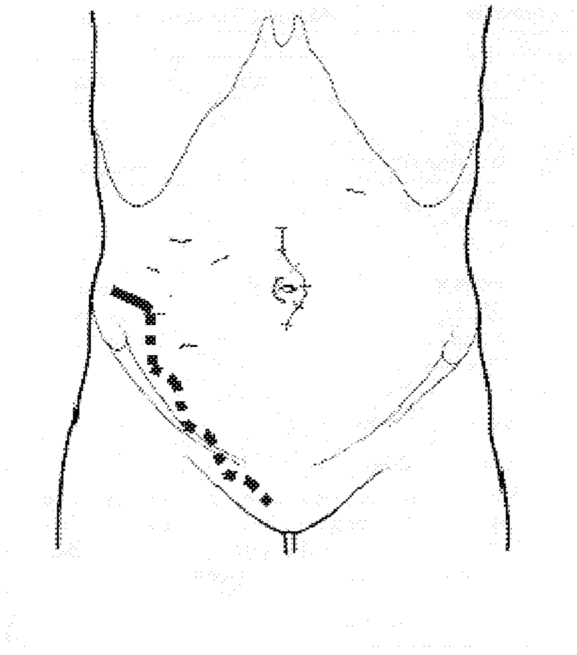

  - colon & rectum

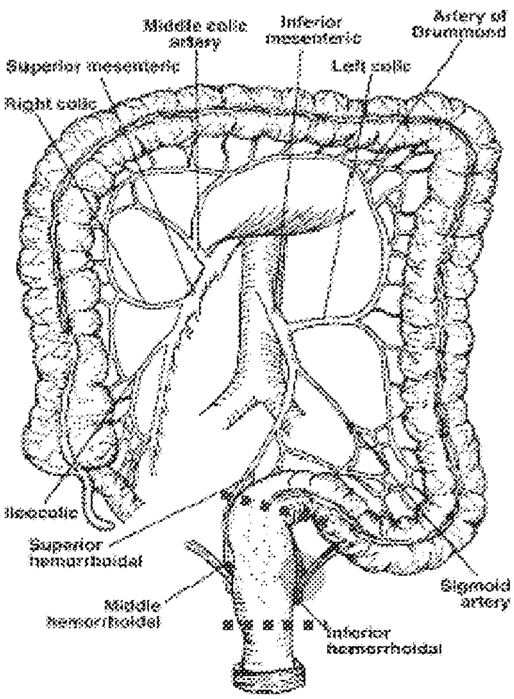

- The resected rectum

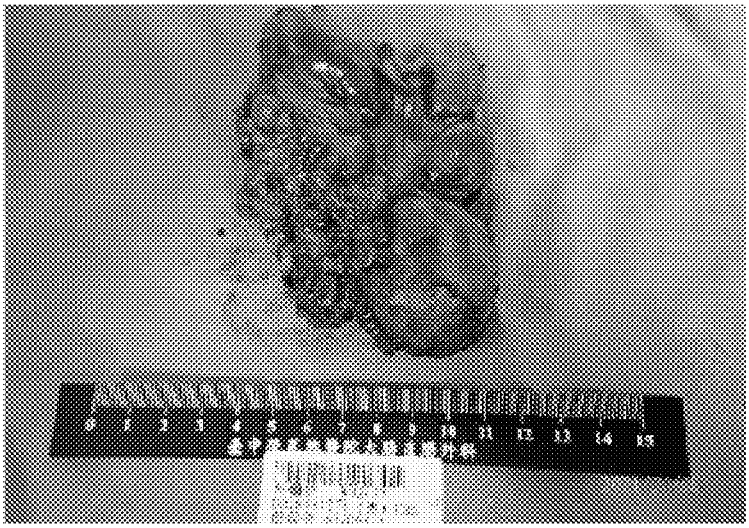

The suspiicous para-rectal mass

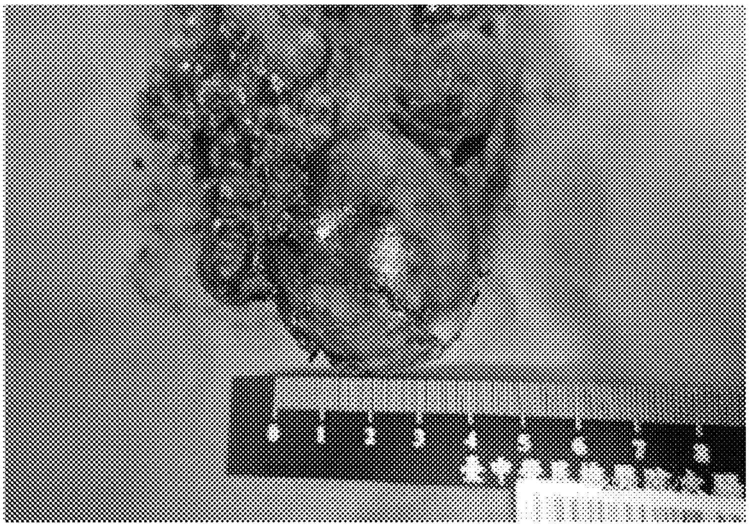

主任 2062F蔣鋒帆 / 醫師 2301H林陳彥

正式摘要**(**電子簽章：**000000214282980)**

請選擇...reporter

doctorRank:B
